# Sesamol promotes browning of white adipocytes to ameliorate obesity by inducing mitochondrial biogenesis and inhibition mitophagy via β3-AR/PKA signaling pathway

**DOI:** 10.29219/fnr.v65.7577

**Published:** 2021-05-10

**Authors:** Cui Lin, Jihua Chen, Minmin Hu, Wenya Zheng, Ziyu Song, Hong Qin

**Affiliations:** Department of Nutrition Science and Food Hygiene, Xiangya School of Public Health, Central South University, Changsha, Hunan, China

**Keywords:** sesamol, obesity, beige adipocytes, mitochondrial biogenesis, mitophagy

## Abstract

**Background:**

Obesity is defined as an imbalance between energy intake and expenditure, and it is a serious risk factor of non-communicable diseases. Recently many studies have shown that promoting browning of white adipose tissue (WAT) to increase energy consumption has a great therapeutic potential for obesity. Sesamol, a lignan from sesame oil, had shown potential beneficial functions on obesity treatment.

**Objective:**

In this study, we used C57BL/6J mice and 3T3-L1 adipocytes to investigate the effects and the fundamental mechanisms of sesamol in enhancing the browning of white adipocytes to ameliorate obesity.

**Methods:**

Sixteen-week-old C57BL/6J male mice were fed high-fat diet (HFD) for 8 weeks to establish the obesity models. Half of the obese mice were administered with sesamol (100 mg/kg body weight [b.w.]/day [d] by gavage for another 8 weeks. Triacylglycerol (TG) and total cholesterol assay kits were used to quantify serum TG and total cholesterol (TC). Oil red O staining was used to detect lipid droplet *in vitro*. Mito-Tracker Green was used to detect the mitochondrial content. Quantitative reverse transcription-polymerase chain reaction (RT-PCR) was used to detect the levels of beige-specific genes. Immunoblotting was used to detect the proteins involved in beige adipocytes formation.

**Results:**

Sesamol decreased the content of body fat and suppressed lipid accumulation in HFD-induced obese mice. In addition, sesamol significantly upregulated uncoupling protein-1 (UCP1) protein in adipose tissue. Further research found that sesamol also significantly activated the browning program in mature 3T3-L1 adipocytes, manifested by the increase in beige-specific genes and proteins. Moreover, sesamol greatly increased mitochondrial biogenesis, as proved by the upregulated protein levels of mitochondrial biogenesis, and the inhibition of the proteins associated with mitophagy. Furthermore, β3-adrenergic receptor (β3-AR), protein kinase A-C (PKA-C) and Phospho-protein kinase A (p-PKA) substrate were elevated by sesamol, and these effects were abolished by the pretreatment of antagonists β3-AR.

**Conclusion:**

Sesamol promoted browning of white adipocytes by inducing mitochondrial biogenesis and inhibiting mitophagy through the β3-AR/PKA pathway. This preclinical data promised the potential to consider sesamol as a metabolic modulator of HFD-induced obesity.

## Popular scientific summary

- Sesamol decreased the content of body fat and suppressed lipid accumulation in high-fat diet-induced mice, and it might be due to promoting white adipocytes browning by sesamol.
- The mitochondrial numbers are known to be correlated with browning of white adipocytes. Sesamol induced white adipocytes browning by promoting mitochondrial biogenesis and inhibiting mitophagy. These effects were associated with the activation of the β3-adrenergic receptor/protein kinase A signaling pathway.
- Promoting white adipocytes browning is emerging as a new strategy to increase energy expenditure and obesity treatment.

Obesity is defined as an imbalance between energy intake and expenditure, and it is a serious risk factor of non-communicable diseases. Presently, the treatments of obesity mainly include dietary intervention, drug intervention and bariatric surgery, but these methods also have limitations, such as insomnia, dysgeusia, irritability, and mood changes (1–3). Therefore, it is needed to develop safe and effective methods for treating obesity.

Recently many studies have shown that promoting browning of white adipose tissue (WAT) to increase energy consumption has a great therapeutic potential for obesity (4, 5). The white adipocyte can be transformed into ‘brown-like’ adipocyte in response to some external stimuli, such as exercise, chronic cold exposure, and agonists of peroxisome proliferator-activated receptor γ (PPARγ) or β3-adrenergic receptor (β3-AR) (6–9). The appearance of inducible brown adipocytes in WAT is defined as beige adipocyte, which is enriched of mitochondria with uncoupling protein-1 (UCP1) to dissipate energy as the brown adipocyte (10–12). Mitochondrial biogenesis and mitophagy are two pathways that regulate mitochondrial content in beige adipocyte. PPARγ coactivator 1α (PGC-1α) is a major regulator of mitochondrial biogenesis. Activated PGC 1α can enhance the mitochondrial biogenesis and energy utilization (13, 14). In addition, inhibited PTEN-induced putative kinase 1 (PINK1) and Parkin could decrease the mitophagy (15). Therefore, if mitochondrial biogenesis got increased while mitophagy could be inhibited, it would be an ideal strategy for increasing beige adipocyte to promote energy consumption and treat obesity (16–18). Recent researches have shown that the β3-AR and its downstream PKA could drive the ‘browning’ effect in WAT, and some studies demonstrated that the activation of PKA would lead to promote mitochondrial biogenesis or inhibit mitophagy (19, 20).

Recent studies have indicated that sesamol, a lignan from sesame oil, had shown potential beneficial functions in obesity treatment (21–24). Our previous research demonstrated that sesamol could attenuate obesity-associated metabolic disorders by activating the PKA pathway in liver (25). Apart from this, there were some observations that sesamol could upgrade the cognitive function by inhibiting the production of amyloid protein in central nervous system of mice (26). Meanwhile, certain reports revealed that activated β-ARs could prevent neuropathy (27, 28). However, the effect of sesamol on regulating β-ARs is ambiguous. Hence, we postulate that sesamol could activate β3-AR and PKA to increase mitochondrial biogenesis and inhibit mitophagy in adipose. In this study, we used C57BL/6J mice and 3T3-L1 adipocytes to investigate the effects and fundamental mechanisms of sesamol that will help in enhancing the browning of white adipocytes to ameliorate obesity.

## Materials and methods

### Chemicals

Sesamol, insulin, dexamethasone, and 3-isobutyl-1-methylxanthine (IBMX) were purchased from Sigma-Aldrich (St. Louis, MO, USA). Triacylglycerol (TG, A110-1-1) and total cholesterol (TC, A111-1-1) assay kits were obtained from Jiancheng Bioengineering Institute (Nanjing, China). The goat anti-rabbit immunoglobulin G (IgG) antibody was purchased from Ding Guo Changsheng Biotechnology Co., Ltd. (Beijing, China). The methyl thiazolyl tetrazolium (MTT) assay kits were obtained from Ding Guo Changsheng Biotechnology Co. Ltd. Oil red O was purchased from Solarbio Science & Technology Co, Ltd. (Beijing, China). Mito-Tracker Green was purchased from Beyotime (Shanghai, China). Antibodies against UCP1 (A5857), PGC-1α (A19674), nuclear respiratory factors 1(NRF1 A5547), PINK1 (A11435), Parkin (A0968), LC3B (A19665), p62 (A19700), β3-AR(A8607), PKA-C (A0798), and β-actin (AC026) were obtained from ABclonal (Boston, MA, USA). Mitochondrial transcription factor A (TFAM AF0531) was bought from Affinity Biosciences (Cincinnati, OH, USA). Phospho-protein kinase A (p-PKA) substrate (9624S) was purchased from Cell Signal Technology (Boston, MA, USA). β3-AR antagonist (SR59230A) was purchased from MedChemExpress (Monmouth Junction, NJ, USA).

### Animals and diet

Eight-week-old male C57BL/6J mice were obtained from Central South University (Hunan, Changsha, China) and maintained under standard laboratory conditions (20–24°C, 40–60% humidity, and light and dark cycle: 12 h/12 h). As previously described (25), the mice were given 1 week to allow them to adapt to the new environment and allowed free access to water and food. Then, one group was fed a normal-fat diet (NFD), forming the control group (*n* = 10), and all other mice received a high-fat diet (HFD group) for 8 weeks to establish the obese model. Then, the HFD group mice whose weights were 20% higher than the average weight of the mice in the control group were further divided into two groups, including the HFD group (*n* = 10) and the HFD + sesamol group (*n* = 10). The HFD group and the HFD + sesamol group continued to receive a HFD, and the control group received an NFD. The HFD + sesamol group were given 100 mg/kg sesamol by gavage for every day, sesamol was dissolved in a vehicle (0.5% carboxylmethylcellulose). Volume and diameter of gavage pipe were, respectively, 1 mL and 45 mm. The control group and the HFD group were given an equal volume of vehicle. The HFD contained 60 kcal% fat, 20 kcal% carbohydrate, and 20 kcal% protein, and the NFD contained 10 kcal% fat, 70 kcal% carbohydrate, and 20 kcal% protein (D12492, Research Diets Inc., New Brunswick, NJ, USA) in this study. Three groups continued to feed for another 8 weeks. Their body weights were measured weekly. The mice were fasted overnight before being sacrificed. Then the body weight was measured, and compositions [inguinal white adipose tissue (iWAT), epididymal WAT (eWAT), and perirenal white adipose (pWAT)] were excised, weighed, and stored at −80°C. The content of body fat (%) was calculated according to the following formula: white adipose weight (g)/body weight (g) × 100%. All animal experiments were performed in accordance with the protocol (Approval Number: XYGW-2019-038) approved by the Institutional Animal Care and Use Committee of Central South University.

### Serum parameter analysis

As previously described (25), after 16 weeks, blood samples were collected from the femoral artery of the mice and stored overnight at 4°C. Then the serum was separated by centrifuging the samples at 3,000 × rpm for 15 min. The serum concentrations of TG and TC were determined using commercially available kits.

### Cell culture and differentiation

3T3-L1 cells were obtained from the Peking Union Cell Center (Beijing, China). As previously described (9), 3T3-L1 cells were cultured in Dulbecco’s modified Eagle medium (DMEM) containing 10% (v/v) newborn calf serum and 1% penicillin–streptomycin solution at 37°C in a humidified atmosphere containing 5% CO_2_. After the cells were grown, the confluent cells were differentiated using DMEM with 10% (v/v) fetal bovine serum (FBS), 8 μg/mL insulin, 1 μM dexamethasone, and 0.5 mM IBMX. After 48 h, the medium was changed to DMEM with 10% (v/v) FBS with 8 μg/mL insulin only for next 48 h. Then, cells were switched to maintenance medium for next 96 h, and the medium was also substituted after every 48 h, so that the mature adipocytes were formed. The mature 3T3-L1 adipocytes were treated with sesamol (12.5, 25 and 50 μM) for 48 h. The cells were then collected for further detection. In the experiments with the β3-AR antagonists, mature 3T3-L1 adipocytes were pretreated with 10 μM SR 59230A 2 h before the 50 μM sesamol treatment.

### Cell viability assay

As previously described (9), 3T3-L1 cells were seeded in a 96-well plate at a density of 5.0 × 10^3^ cells/well and then were incubated until mature adipocytes formed, as discussed earlier. Then, cells were treated with different concentrations of sesamol (0, 12.5, 25, 50, 100, 200, and 400 μM) for 48 h. At the end of the incubation, an amount of 10 μL of MTT solution was added to each well, and the cells were further incubated for 4 h. The media was detached then and upon shaking the 150 μL of dimethyl sulfoxide (DMSO) was added for up to 10 min to reunite the cells. Absorbance was measured at 490 nm using a microplate reader (Multiskan Sky Microplate Spectrophotometer, Thermo Fisher Scientific, MA, USA).

### Oil red O staining

Intracellular lipid accumulation was quantified by staining with oil red O. The mature cells that were treated with sesamol for 48 h were washed twice with phosphate-buffered saline (PBS), fixed with 4% paraformaldehyde for 10 min, and finally stained with oil red O working solution for 30 min. After the staining solution was removed, the cells were washed with distilled water. The stained lipid droplets were envisioned under an inverted microscope.

### Mitochondrial content assay

The mature cells were treated with sesamol for 48 h. The mitochondrial content in mature 3T3-L1 adipocytes was determined by Mito-Tracker Green according to the manufacture’s procedure. Fluorescent images were captured with an Invitrogen Imaging System. Relative fluorescence intensity of mitochondria was measured in three fields per sample using ImageJ software.

### Quantitative reverse transcription-polymerase chain reaction

Total RNAs were isolated from mature white cells and were treated with sesamol for 48 h. RNA (1 μg) was converted to cDNA using Hifair II 1st cDNA Synthesis SuperMix for quantitative polymerase chain reaction (qPCR, YEASEN, Shanghai, China). Hieff qPCR SYBR Green Master Mix (YEASEN, Shanghai, China) was engaged to computably decide transcription levels of genes with quantitative reverse transcription-polymerase chain reaction (RT-PCR) (LightCycler 480 II, Roche, Basel, Switzerland). The cycling conditions were as follows: 95°C for 3 min, followed by 40 cycles of 95°C for 10 sec and 60°C for 30 sec. The melting curve was also examined to ensure that only a solitary product was magnified, and the conditions were as follows: 95°C for 15 sec, 60°C for 1 min, and 95°C for 15 sec. PCR reactions were travelled in threefolds for each and every sample, and the relative mRNA expression was evaluated after the values become normal as that of β actin. Sequences of primer sets used in this study are listed in Table 1.

**Table 1:** Primer sequences

Gene Name	Primer Sequence (5’to 3’)		Ref.
	Forward	Reverse	
*Ucp1*	ACT GCC ACA CCT CCA GTC ATT	CTT TGC CTC ACT CAG GAT TGG	(29)
*Cd40*	GCTATGGGGCTGCTTGTT	GGGTGGCATTGGGTCTTC	(9)
*Temem26*	TGCCAGGAAGTCAGAGAG	CAAAGCAGCCAGCATAAG	(9)
*β-actin*	TGCGTTTTACACCCTTTC	CTGTCGCCTTCACCGTTC	

### Immunoblotting

iWAT (20 mg) was homogenized in 400 μL radioimmunoprecipitation assay (RIPA) buffer with a protease inhibitor. The tissue was homogenized using a rotor-stator homogenizer on ice for 1 min and then centrifuged at 15,000 rpm for 15 min at 4°C. The mature 3T3-L1 adipocytes that were treated with sesamol for 48 h were harvested with RIPA buffer containing protease inhibitors. Protein contents were determined by BCA assay kits. Then, the proteins (40 μg) were separated by 10–12% or 15% sodium dodecyl sulfate polyacrylamide gel electrophoresis (SDS–PAGE) and transferred to polyvinylidene fluoride (PVDF) membranes. After that when the membranes were blocked with 5% skim milk or 3% bovine serum albumin (BSA) in Tris-buffered saline and Tween 20 (TBST) at room temperature for 1 h, they were incubated with primary antibody overnight at 4°C specific to β3-AR (1:1,000), PGC-1α (1:1,000), UCP1 (1:1,000), NRF1(1:1,000), (TFAM) (1:750), PINK1 (1:1,000), Parkin (1:1,000), p62 (1:1,000), LC3B (1:1,000), and PKA-C (1:1,000). Membranes were then washed in TBST and after that incubated with the goat anti-rabbit IgG antibody (1:5,000) for 1 h at room temperature. The signal was detected easily by using the chemiluminescence imager (Tanon-5500, Tanon Science & Technology Co., Ltd., Shanghai, China). The relative expression of proteins was quantified by Tanon Gis software and was calculated according to the reference bands of β-actin (1:300,000). Immunoblotting analyses represent three independent experiments.

### Statistical analysis

All statistical analyses were performed using SPSS 20.0 software (SPSS, Inc., Chicago, IL, USA). All quantitative data were reported as mean ± standard deviation (SD). Differences among groups were quantified by one-way analysis of variance (ANOVA). Significant differences between the mean values were assessed using least-significant difference t-test (LSD-t). Level of significance was set at 95% (*P* < 0.05).

## Results

### Sesamol reduced the content of body fat and suppressed lipid accumulation in HFD- induced mice

**Fig. 1:**
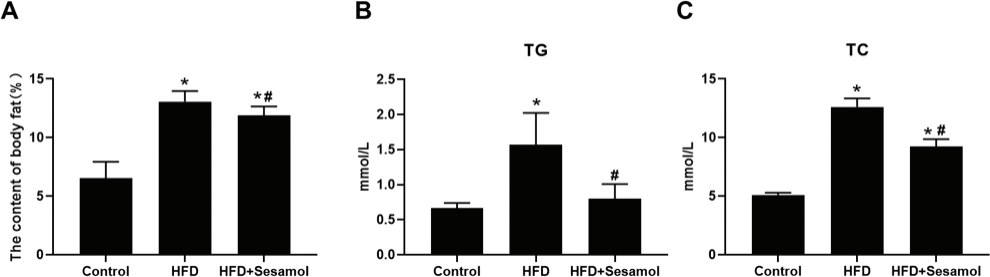
Effects of sesamol on the content of body fat and lipid accumulation in HFD-induced mice. (A) the content of body fat (*n* = 10), (B) the serum TG levels, and (C) the serum TC levels (*n* = 3). All values are presented as mean ± SD. *P < 0.05 versus control group, #*P* < 0.05 versus HFD group.

To explore the effect of sesamol in ameliorating obesity, HFD-induced obese mice were administered sesamol by gavage for 8 weeks. We found that the content of body fat was decreased after sesamol intervention in HFD- induced obese mice (Fig. 1A). We next investigated the effects of sesamol on the serum TG and TC of the mice. We found that serum TG and TC levels were reduced after sesamol intervention (Fig. 1B and C). Taken together, these data indicated that sesamol intervention ameliorated HFD-induced obesity and suppressed lipid accumulation.

### Sesamol upregulated UCP1 protein level in white adipose of HFD-induced obese mice

**Fig. 2:**
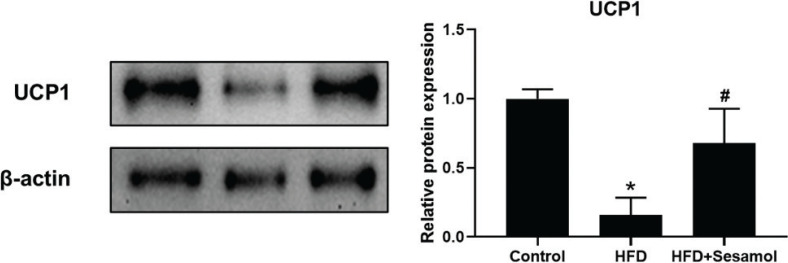
Effect of sesamol on UCP1 protein level in white adipose of HFD-induced mice. Immunoblotting was used to detect protein expression of UCP1 and densitometric determinations. β-actin and histone were protein loading control. All values are presented as mean ± SD (*n* = 3). *P < 0.05 versus control group, #*P* < 0.05 versus HFD group.

We sought to measure whether amelioration of obesity was associated with the browning of adipose tissue. We found that after sesamol intervention the UCP1 protein levels were increased in white adipose of HFD-induced obese mice (Fig. 2). UCP1 is one of the beige fat-specific markers, and its expression allows for transport of protons back across the inner mitochondrial membrane, uncoupling oxidative phosphorylation from ATP production and resulting in thermogenesis. This result showed that sesamol promoted white adipose browning in HFD-induced mice.

### Effects of sesamol on viability of mature 3T3-L1 adipocytes

**Fig. 3:**
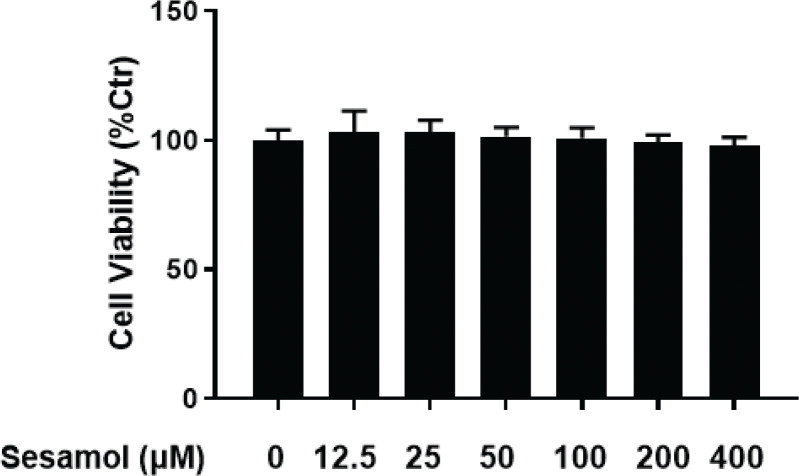
Effect of sesamol on mature 3T3-L1 adipocyte viability. The mature adipocytes were treated with different concentrations of sesamol (0, 12.5, 25, 50, 100, 200, and 400 μM for 48 h, and viability was determined by MTT assay. Values are presented as mean ± SD (*n* = 5).

The cytotoxic effect of sesamol on mature 3T3-L1 adipocytes was examined by MTT assay at different doses. The results showed that there was no significant decrease in cell viability after sesamol intervention (Fig. 3). Based on these results, 12.5, 25, and 50 μM were chosen for the succeeding experiments.

### Sesamol increased the expressions of beige-specific markers in mature 3T3-L1 adipocytes

**Fig. 4:**
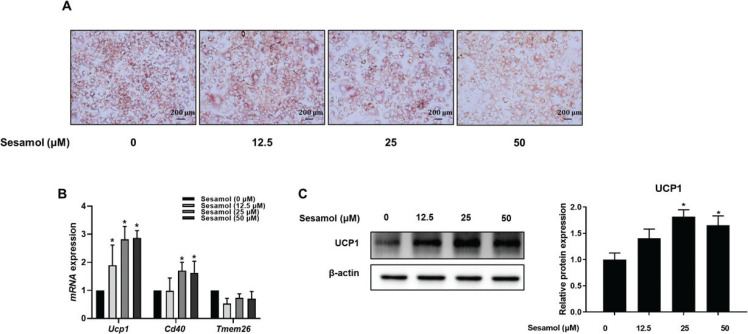
Effects of sesamol on lipid accumulation and expressions of beige-specific genes and proteins in mature 3T3-L1 adipocytes. The mature 3T3-L1 adipocytes were treated with different concentrations of sesamol (0, 12.5, 25, and 50 μΜ) for 48 h. (A) Mature 3T3-L1 adipocytes were stained with oil red O to observe lipid droplets at 200 × magnification. Scale bar = 200 μm. (B) RT-PCR was used to detect the levels of beige-specific genes. (C) Immunoblotting was used to detect protein expression of UCP1 and densitometric determinations. All values are presented as mean ± SD (*n* = 3). β-actin and histone were protein loading control. *P < 0.05 versus control group (0 μM sesamol).

Compared with the control group, a decrease in lipid droplets was recorded as a result of sesamol treatment in mature 3T3-L1 adipocytes (Fig. 4A). The reason was that the beige adipocytes could burn lipid to dissipate energy in the form of heat (10). We sought to further investigate if the effect of sesamol on the reduction of lipid droplets was related to the induction of beige formation. Hence, we analyzed the gene and protein levels of the beige markers, and found that the genes (*Cd40* and *Ucp1*) and the protein UCP1 were significantly increased after sesamol treatment (Fig. 4B and C). These results demonstrated that sesamol could reduce lipid accumulation by inducing beige adipocytes formation.

### Sesamol increased mitochondrial numbers in mature 3T3-L1 adipocytes

**Fig. 5:**
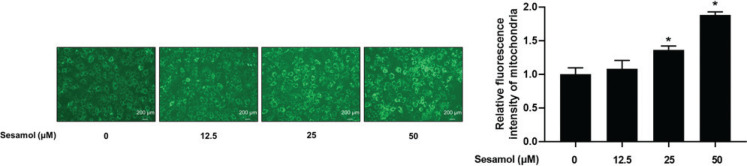
Effect of sesamol on mitochondrial numbers in mature 3T3-L1 adipocytes. The mature 3T3-L1 adipocytes were treated with 12.5, 25, and 50 μM sesamol for 48 h. Cells were stained with mito-tracker Green to observe mitochondrial numbers at 200 × magnification. Scale bar = 200 μm. All values are presented as mean ± SD (*n* = 3). *P < 0.05 versus control group (0 μM sesamol).

Mitochondrion is one of the most important indicative organelles of beige formation. We used Mito-Tracker Green to observe whether sesamol could increase mitochondrial numbers. We found that the fluorescence intensities were increased after sesamol intervention in mature 3T3-L1 adipocytes (Fig. 5). The results showed that sesamol could induce white adipocytes browning by increasing mitochondrial numbers.

### Sesamol promoted mitochondrial biogenesis in mature 3T3-L1 adipocytes

**Fig. 6:**
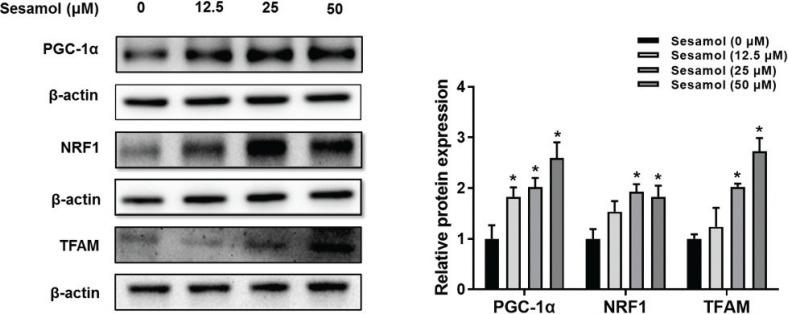
Effects of sesamol on the expressions of mitochondrial biogenesis proteins in mature 3T3-L1 adipocytes. The mature 3T3-L1 adipocytes were treated with different concentrations of sesamol (0, 12.5, 25, and 50 μM) for 48 h. Immunoblotting was used to detect protein expression of PGC-1α, NRF1, TFAM, and densitometric determinations. All values are presented as mean ± SD (*n* = 3). β-actin and histone were protein loading control. *P < 0.05 versus control group.

Mitochondrial number is regulated by mitochondrial biogenesis (30). We found that sesamol dose-dependently increased the level of PGC-1α, NRF1, and TFAM (Fig. 6). These results revealed that sesamol could increase the mitochondrial number by promoting mitochondrial biogenesis.

### Sesamol inhibited mitophagy in mature 3T3-L1 adipocytes

**Fig. 7:**
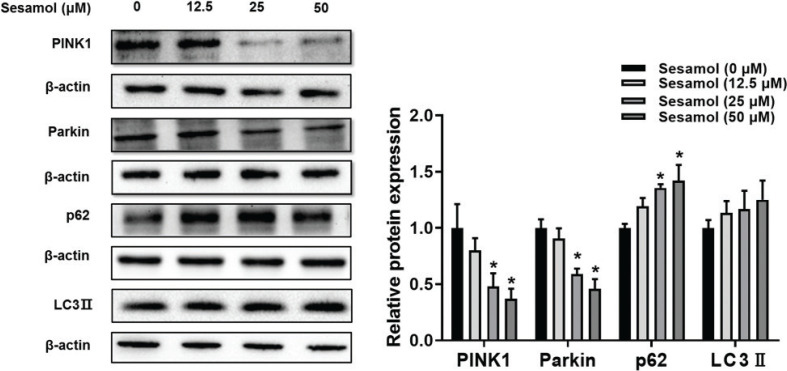
Effects of sesamol on the expressions of mitophagy proteins in mature 3T3-L1 adipocytes. The mature 3T3-L1 adipocytes were treated with different concentrations of sesamol (0, 12.5, 25, and 50 μM). Immunoblotting was used to detect protein expression of PINK1, Parkin, p62, LC3 II, and densitometric determinations. All values are presented as mean ± SD (*n* = 3). β-actin and histone were protein loading control. *P < 0.05 versus control group.

Mitophagy was another factor that affected the mitochondrial numbers (31). We found that sesamol significantly reduced the protein levels that related to mitophagy, such as PINK1 and Parkin (Fig. 7). LC3 is an essential component of autophagosomes, which is widely used as an autophagy marker (32). But currently, the protein level of LC3 II was not changed after sesamol treatment, while another autophagy marker, p62, was increased (Fig. 7). p62 can be used to detect the level of autophagy (33). The increase in p62 level indicated that autophagy is inhibited, but a decrease in p62 level showed that autophagy is induced (32, 34). Hence, the increase of p62 level that was observed in our study showed that mitophagy is inhibited. These results indicated that sesamol could inhibit mitophagy to induce browning of white adipocytes.

### Sesamol induced white adipocytes browning by β3-AR/PKA signal pathway

**Fig. 8:**
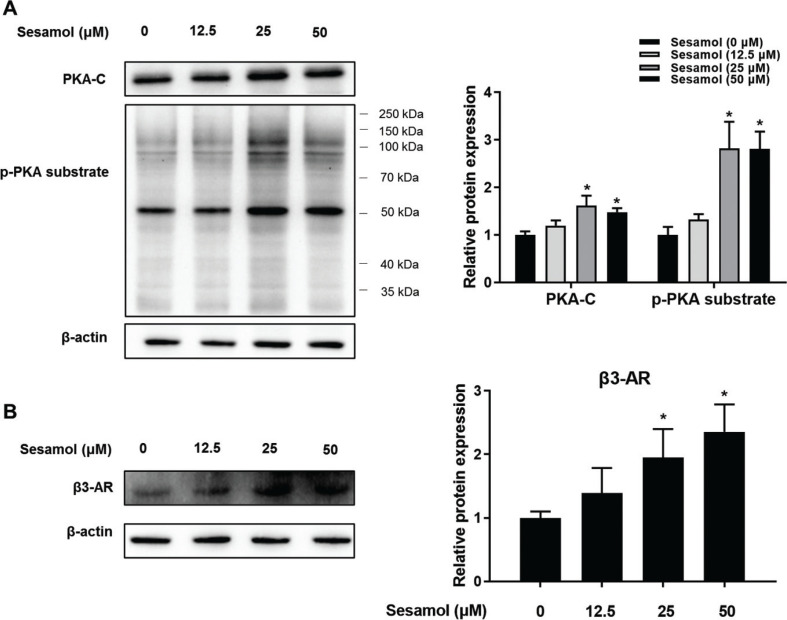
Effects of sesamol on the expressions of mitophagy proteins in mature 3T3-L1 adipocytes. The mature 3T3-L1 adipocytes were treated with different concentrations of sesamol (0, 12.5, 25, and 50 μΜ) for 48 h. Immunoblotting was used to detect protein expression. (A) PKA-C, p-PKA substrate levels, and densitometric determinations. (B) β3-AR levels and densitometric determinations. All values are presented as mean ± SD (*n* = 3). β-actin and histone were protein loading control. *P < 0.05 versus control group.

**Fig. 9:**
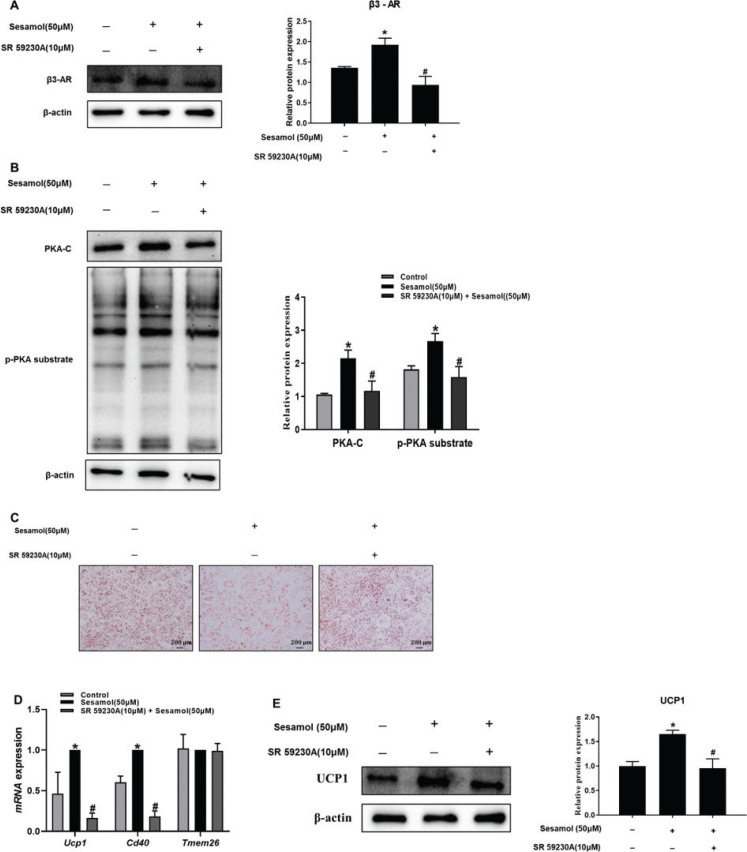
Effects of SR 59230A on the expressions of beige-specific genes and proteins in mature 3T3-L1 adipocytes treated with sesamol. The mature 3T3-L1 adipocytes were pretreated with 10 μM SR 59230A for 2 h prior to sesamol (50 μM) treatment. RT-PCR was used to detect the genes level and immunoblotting was used to detect protein expression. (A) β3-AR levels and densitometric determinations. (B) PKA-C, p-PKA substrate levels, and densitometric determinations. (C) Mature 3T3-L1 adipocytes were stained with oil red O to observe lipid droplets at 200 × magnification. (D) The levels of beige-specific genes. (E) Protein expression of UCP1 and densitometric determinations. All values are presented as mean ± SD (*n* = 3). β-actin and histone were protein loading control. *P < 0.05 versus the control group; #*P* < 0.05 versus the sesamol group.

PKA is the key factor both in the mitochondrial biogenesis and mitophagy pathway (31, 35). To test the effect of sesamol on PKA, we measured the expression of PKA-C and p-PKA substrates and found that the protein levels of PKA-C and p-PKA substrate were remarkably increased (Fig. 8A). β3-AR is one of the important regulators for adipocyte browning, and is crucial regulator of PKA (36). Our results showed that the β3-AR protein levels were dose dependent and regulated up by sesamol (Fig. 8B). To further confirm the role of β3-AR and PKA in moderating the effects of sesamol, we pretreated the mature 3T3-L1 adipocytes with the inhibitors of β3-AR (SR59230A). We found that the increases in β3-AR, PKA-C, and p-PKA substrate level were eliminated by the pretreatment of SR59230A (Fig. 9A and B). In addition, we also detected that the effects on the decrease in lipid droplets and increase in beige adipocytes marker genes (*Ucp1*and *Cd4*) and protein (UCP1) level were blocked by inhibited β3-AR activity (Fig. 9C–E). These results suggested that sesamol could induce white adipocytes browning by activating β3-AR/PKA pathway.

## Discussion

In this study, we found that sesamol could decrease the content of body fat and suppress lipid accumulation by inducing browning of white adipose of HFD-induced mice. At the molecular level, we first demonstrated that sesamol induced white adipocytes browning by promoting mitochondrial biogenesis and inhibiting mitophagy. These effects were associated with the activation of the β3-AR/PKA signaling pathway.

Obesity is one of the global public health concerns. Obesity is characterized by increased adipose tissue mass, and it is associated with a greater susceptibility to metabolic diseases (37, 38). Adipose tissues are one of the most important components for regulating the energy metabolism. White adipose is specialized to store energy in the form of triglycerides, but beige adipocytes, which are converse of white adipocytes, have the specialty to spend energy through heat production, because they possess more mitochondria than white adipocytes (39–41). Therefore, the browning of white adipocytes has the potential to treat the obesity problem.

The current study found that sesamol treatment decreased the content of body fat and reduced serum TG and TC levels in HFD-induced mice. We also found that the lipid droplets in mature 3T3-L1 adipocytes decreased after sesamol intervention. These results showed that sesamol could ameliorate obesity and reduce lipid accumulation *in vivo* and *in vitro*. Moreover, our data showed that sesamol treatment upregulated the UCP1 level in WATs of HFD-induced mice and mature 3T3-L1 adipocyte. UCP1 is a mitochondrial membrane protein, and its expression allows for transport of protons back across the inner mitochondrial membrane, uncoupling oxidative phosphorylation from ATP production and resulting in thermogenesis (42). In addition, the gene markers of beige adipocyte increased after sesamol treatment, such as *Ucp1* and *Cd40* in mature 3T3-L1 adipocytes. These findings indicated that sesamol promoted the browning of white adipocytes. Taken together, these data showed that sesamol could ameliorate obesity through inducing beige adipocytes formation. Since the increase in mitochondria is an important factor that distinguishes beige adipocytes from white adipocytes, we used immunofluorescence to detect the mitochondrial numbers, and found that after sesamol treatment the mitochondrial number increased in a dose-dependent manner. These results demonstrated that sesamol encouraged browning of white adipocytes correlated with the rise in mitochondrial number.

Mitochondrial number could be regulated by mitochondrial biogenesis and mitophagy separately (31). We further inspected whether sesamol regulates these two biological processes. First, we found that the protein expression of PGC-1α in mature 3T3-L1 adipocyte enhanced in a dose-dependent manner after the sesamol treatment. PGC-1α is the key regulator of mitochondrial biogenesis. It could induce the expression of nuclear respiratory factors including NRF1 and NRF2, which would upregulate the mitochondrial biogenesis (43, 44). Moreover, NRF1 and NRF2 directly interacted with TFAM to enhance transcription and replication of mitochondrial DNA (mtDNA) (45, 46). Our result revealed that sesamol enhanced the protein expression of NRF1 and TFAM to increase the mitochondrial biogenesis. Second, we also observed that sesamol could decrease the protein level of PINK1, which is one of the important factors for mitophagy. Recent studies have shown that the beige adipocytes recuperated to white adipocytes after stimuli, and the change of beige-to-white adipocyte transition was relative to mitophagy (16, 47, 48). PINK1 is present in the outer membrane of mitochondria. When mitochondrial membrane potential gets decreased, PINK1 signs up the Parkin proteins from the cytosol to the mitochondria (49, 50). Later on, p62 linked the ubiquitinated mitochondrial proteins to LC3, leading to mitophagy (51, 52). Therefore, we examined whether the PINK1 regulated proteins were associated with mitophagy. We found that the expression of Parkin proteins was decreased after sesamol treatment with the adipocytes. Although the expression of LC3 was not changed, the expression of p62 was increased. LC3 is a marker that represents a change in the number of autophagosomes (32, 53). In the present study, the level of LC3-II was not changed, which indicated that the autophagosome clearance was not changed. However, an increase in p62 level showed that autophagic flux *per se* was blocked. Taken together, these findings indicate that mitophagy was inhibited after the sesamol treatment. These results demonstrated that sesamol inhibited mitophagy to maintain the beige adipocytes.

Generally, β3-AR can increase the level of cAMP, leading to the activation of PKA (36). It is known that PKA is an upstream kinase of PGC-1α, and the activation of PKA can decrease the level of PINK1 and Parkin (16–18). Therefore, we conjectured that it was possible that sesamol activated β3-AR/PKA signaling pathway to regulate mitochondrial biogenesis and mitophagy. As anticipated, our results indicated that sesamol treatment dose-dependently increased the PKA-C and p-PKA substrate levels. Our data also confirmed that sesamol intervention increased β3-AR level. Furthermore, to assess the effects of sesamol on browning of white adipocytes and mitochondrial level were due to the activation of β3-AR/PKA signaling pathway, we administered the regulation of downstream signals of β3-AR in mature 3T3-L1 adipocytes in the presence of β3-AR inhibitor, SR 59230A. We found that the effects of sesamol on promoting browning of white adipocytes were eradicated. These results demonstrated that sesamol could increase mitochondrial biogenesis and inhibit mitophagy simultaneously to enhance browning of white adipocytes by directly enhancing the β3-AR/PKA signaling pathway.

However, obesity can develop in many other ways than through HFD alone; therefore, in the future we would further investigate whether sesamol could ameliorate the obesity caused by other factors, such as high-sucrose diet-induced obesity or genetic obesity. In addition, although animal experiment can provide scientific evidence for the application of sesamol, it does not precisely reflect the states in human beings. In future study, we would perform experiments by using multipotent human adipose-derived stromal progenitor cells, and eventually verify the effects of sesamol through clinical experiments.

## Conclusion

**Fig. 10:**
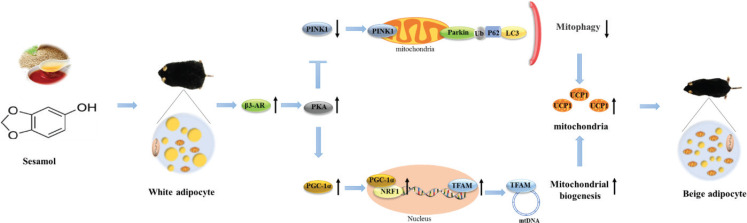
Sesamol promotes browning of white adipocytes to ameliorate obesity by inducing mitochondrial biogenesis and inhibition mitophagy via *β*3-AR/PKA signaling pathway.

For the first time, the data of our study indicated that sesamol promoted browning of white adipocytes by inducing mitochondrial biogenesis and inhibiting mitophagy through the β3-AR/PKA pathway. This pre-clinical data promised the potential to consider sesamol as a metabolic modulator of HFD-induced obesity (Fig. 10).
